# Neonatal outcomes from a quasi-experimental clinical trial of Family Integrated Care versus Family-Centered Care for preterm infants in U.S. NICUs

**DOI:** 10.1186/s12887-022-03732-1

**Published:** 2022-11-22

**Authors:** Linda S. Franck, Caryl L. Gay, Thomas J. Hoffmann, Rebecca M. Kriz, Robin Bisgaard, Diana M. Cormier, Priscilla Joe, Brittany Lothe, Yao Sun

**Affiliations:** 1 Department of Family Health Care Nursing, University of California San Francisco (UCSF), Box 0606, 2 Koret Way, N411F, CA 94143 San Francisco, USA; 2 Department of Epidemiology and Biostatistics, Office of Research, School of Nursing, UCSF, San Francisco, CA USA; 3 Intensive Care Nursery, UCSF Benioff Children’s Hospital, San Francisco, CA USA; 4 NICU and Pediatrics, Community Regional Medical Center, Fresno, CA USA; 5 Division of Neonatology, UCSF Benioff Children’s Hospital, Oakland, CA USA; 6 Will’s Way Foundation, Chicago, IL USA; 7 Division of Neonatology, Department of Pediatrics, UCSF, San Francisco, CA USA

**Keywords:** Family partnerships, Infant, Neonatology, Weight gain, Nosocomial infection, Peer mentors, Clinical rounds, Parent education

## Abstract

**Background:**

Family Integrated Care (FICare) benefits preterm infants compared with Family-Centered Care (FCC), but research is lacking in United States (US) Neonatal Intensive Care Units (NICUs). The outcomes for infants of implementing FICare in the US are unknown given differences in parental leave benefits and health care delivery between the US and other countries where FICare is used. We compared preterm weight and discharge outcomes between FCC and mobile-enhanced FICare (mFICare) in the US.

**Methods:**

In this quasi-experimental study, we enrolled preterm infant (≤ 33 weeks)/parent dyads from 3 NICUs into sequential cohorts: FCC or mFICare. Our primary outcome was 21-day change in weight z-scores. Our secondary outcomes were nosocomial infection, bronchopulmonary dysplasia (BPD), retinopathy of prematurity (ROP), and human milk feeding (HMF) at discharge. We used intention-to-treat analyses to examine the effect of the FCC and mFICare models overall and per protocol analyses to examine the effects of the mFICare intervention components.

**Findings:**

253 infant/parent dyads participated (141 FCC; 112 mFICare). There were no parent-related adverse events in either group. In intention-to-treat analyses, we found no group differences in weight, ROP, BPD or HMF. The FCC cohort had 2.6-times (95% CI: 1.0, 6.7) higher odds of nosocomial infection than the mFICare cohort. In per-protocol analyses, we found that infants whose parents did not receive parent mentoring or participate in rounds lost more weight relative to age-based norms (group-difference=-0.128, CI: -0.227, -0.030; group-difference=-0.084, CI: -0.154, -0.015, respectively). Infants whose parents did not participate in rounds or group education had 2.9-times (CI: 1.0, 9.1) and 3.8-times (CI: 1.2, 14.3) higher odds of nosocomial infection, respectively.

**Conclusion:**

We found indications that mFICare may have direct benefits on infant outcomes such as weight gain and nosocomial infection. Future studies using implementation science designs are needed to optimize intervention delivery and determine acute and long-term infant and family outcomes.

**Clinical Trial Registration:**

NCT03418870 01/02/2018.

## Introduction

Parent contributions to the care and outcomes of preterm infants admitted to Neonatal Intensive Care Units (NICU) is undisputed [1–4]. Family-centered care (FCC) is a term used to describe a set of principles that assert the central role families (parents or other primary caregivers) have in promoting the health and development of critically ill infants receiving intensive care and guide the relationship between healthcare professionals and family caregivers [3]. FCC is also an umbrella term used to describe models of NICU care where parents are active partners in shared decision-making and direct caregiving for their infant [1]. NICUs with care delivery models based on FCC principles promote specific parent-delivered interventions, such as breastfeeding, skin-to-skin contact, developmentally-supportive care, positive sensory stimulation, pain management and massage. These practices lead to improved outcomes for preterm infants and families [1]. However, full parental partnership care and decision-making remains an elusive goal in most NICUs [1, 5]. It also remains unclear what ‘dose’ of FCC practices and parental involvement are necessary for optimal infant outcomes. Moreover, there are likely disparities in parental involvement based on parental and NICU resources [6].

The Family-Integrated Care (FICare) model has emerged as a well-defined yet flexible model of parent-partnered NICU care that has been shown to improve infant and parent outcomes in clinical trials and quality improvement evaluations across high- and middle-income countries and levels of neonatal care [7–15]. FICare has four main pillars: NICU environment, NICU team education and support, parent education, and parent support [16]. Clinical trials have demonstrated improved weight gain [9–11], breastfeeding at discharge [9–11], shorter lengths of stay [9, 10, 12, 13], and lower rates of sepsis [10, 13] for infants in FICare versus FCC NICUs. Longer-term outcomes include better mental and psychomotor development at 18 months for infants from FICare versus FCC NICUs [8, 10, 14].

There are fundamental differences between United States (US) health and social care systems and those of other countries that might impact FICare feasibility and outcomes. Families in the United States (US) have less access to statutory paid parental leave benefits than most countries [17], severely curtailing their participation in caregiving during prolonged NICU hospitalization. US families also may have greater healthcare administrative and cost burden (e.g., obtaining information, referrals, insurance authorizations, billing issues, and out-of-pocket expenses) [18]. Notably, the published FICare research to date has been conducted outside the US.

Mobile technology has been proposed to enhance FICare delivery by providing parents with greater access to educational content and encouragement, as well as promoting partnership between parents and NICU staff [12]. Mobile technology may also aid in the research process by improving participant data collection efficiency and experience. Therefore, we aimed to investigate the effects of a mobile-enhanced FICare (mFICare) program in US NICUs. We hypothesized that infants whose parents participated in mFICare would have greater weight gain and fewer morbidities than infants whose parents received usual FCC. We also examined mFICare program components to determine their differential effects on infant outcomes.

## Methods

### Design

In planning for this study, we first engaged extensively with parents of current and former NICU patients and with NICU healthcare professionals to tailor the FICare intervention to the local setting and to develop and pilot a mobile app for parents [19]. We then conducted a quasi-experimental, time-lagged intervention trial (NCT03418870; 01/02/2018), in which we prospectively enrolled infant/parent dyads into one of two sequential cohorts. This design was chosen because it was impossible to individually randomize the intervention without significant risk of spillover and contamination of the study groups. The first cohort included six sites where all infants and families received usual FCC. After completion of enrolment of the FCC cohort, we paused recruitment for mFICare training of NICU staff and parent mentors. Once training was completed, we enrolled the second cohort of parent/infant dyads to participate in the mFICare program because only three sites secured funding to participate in the mFICare cohort. This analysis compares infant outcome for the usual FCC and mFICare cohorts at those three sites.

### Sample size estimate

The sample size for our main infant outcome, change in weight z-score, was established for our primary site, and other sites were experimental and included for feasibility and acceptability evaluation and to increase representativeness [19]. For the primary site, with a projected 100 participants, 20% attrition (80 participants after attrition), a standard deviation in infant weight z-scores of 0.44–0.47 [11], and a two-tailed t-test with conventional alpha 0.05, we estimated 80% power to detect a group difference of 0.28 to 0.29. Since we included the additional sites in our analysis, and adjusted for site, we expected to be able to detect a slightly smaller effect size than this.

### Setting

The three sites included one level IV NICU with single and double family rooms in a university health system (Site A), one level IV NICU in a free-standing children’s hospital with three large open-bay rooms (Site B), and one large level III NICU in a community hospital with many small and large open bay rooms across 2 floors (Site C). All NICUs were regional centers providing care to infants from ethnically diverse urban and rural communities. Sites A and C provided high-risk maternity care with NICUs serving both inborn and outborn neonates, whereas Site B served outborn neonates only. Sites B and C primarily served neonates whose families had publicly funded health insurance or were without coverage, whereas Site A served more families with private health insurance. The NICUs provided FCC as their standard model of NICU care and encouraged 24/7 parental/primary caregiver presence.

### Participants

Parents/primary caregivers of infants born at ≤ 33 weeks gestation were invited to participate and give consent for their infant’s participation. Parent/infant dyads were excluded if: (1) the parent was not English literate, < 18 years of age, or had no smart phone or tablet access; or (2) the infant had a life-threatening congenital anomaly or was receiving palliative care. For multiple births, the primary parent self-identified as spending the most time in the NICU and the primary infant was selected by random assignment. If a second parent met eligibility criteria and wanted to participate, they were assigned the second infant (by random assignment if triplets or quadruplets). Parents of eligible infants were approached about study enrollment early in the NICU admission, but given that the initial days and sometimes weeks of a NICU stay are typically a stressful time for families, they also had the option to defer until a later time, as long as the infant was expected to remain in the NICU for a minimum of 21 days. In addition, outborn infants may have been enrolled later, depending on their age at admission to the study site. Parents received up to $50 in gift cards for completion of study surveys. The study was approved by the institutional review boards at each site and written informed consent was obtained from all parents.

### Intervention

Details of usual FCC and mFICare model components are provided in Table 1. Briefly, parents/infants enrolled in the FCC group received standard NICU care that included a supportive physical and interpersonal environment. Parents were encouraged to spend extended periods in the NICU with their infant. The environment varied by site, but all included reclining chairs, family lounges, kitchens, locked personal storage, Wi-Fi access, and breast pumps. All sites provided NICU orientation for parents and written and video materials to support parent knowledge, skill-building, and coping. Individualized parent teaching and support were delivered bedside by nurses, discharge coordinators, or other specialists, and in a discharge class (sites B and C). Parents were encouraged to participate in infant care under nursing supervision for feeding, bathing, dressing, and holding skin-to-skin. Individualized support was also provided by social workers, developmental specialists, lactation consultants, physical therapy, occupational therapy, or other specialists. Participants also received instruction on using We3health™ Tracker, an app co-designed by parents to document time spent with their infant, infant caregiving, observations, learning needs and skills acquisition.

**Table 1:** Comparison of Family-Centered Care (FCC) and mobile-enhanced Family-Integrated Care (mFICare)

Domain	FCC group	mFICare group
NICU staff education	• Variable, individually-motivated education; no formal ongoing support	• Formal, unit-wide education and support for nursing role in mFICare; additional education for volunteer nurse “mFICare champions” • Ongoing support and mentoring for nurses by mFICare champions
Parent education/ empowerment	• 1:1 teaching at bedside • Discharge class (sites B and C) • Monthly parent support group (site C)	• 1:1 teaching at bedside • In-person education and peer support group classes with 3-week rotating curriculum offered 2 to 5 times per week at each site (emphasis on developmentally-supportive care, feeding, preparation for discharge) • The small group sessions were facilitated by a member of the study team, clinical staff, or alumni parents • Parents participated in-person or could access the content remotely at a time of their choosing via We3health™
Parental involvement in infant’s care	• Parents encouraged be involved in infant’s basic care	• Parents expected to contribute to infant’s care as much as possible and are supported to do so
Partnership in care planning	• Variable encouragement to be present during rounds • No formal role for parents	• Parents encouraged and supported to participate in weekday clinical team rounds either in-person or remotely via telephone • Parents receive training and role-modeling from their infant’s nurses or mFICare nurse champion to provide a brief report of their infant’s status to the rounding care team, ask questions, and reach consensus with the clinicians on the infant’s daily plan • The level of parent participation increased over time (3 training scripts) from introducing themselves and their infant to the team to providing a more detailed report of their observations and infant's responses to caregiving
Parent peer mentor support	• No formal parent mentor program	• Formal peer support provided by trained peer parent mentors (parents of former NICU patients). Parents were offered peer-to-peer support from alumni parent mentors at sites A and C, and referred to a national NICU parent peer support service at site B. For the local programs, social workers connected parents with a mentor and communication between parents and their mentor occurred through text, email or telephone, with occasional in-person meetings. For the national program, parent-to-parent communication occurred via social media, text, telephone or email.
Mobile app for parents	• Parents were provided with the We3health^™^ Tracker version app and encouraged to document: o Time spent in NICU o Time spent in skin-to-skin care o Time spent breast-feeding/pumping breastmilk o Weekly knowledge needs and skills learned o Experiences and feelings in online text/photo journal	• Parents were provided with the We3health^™^ mFICare version app and encouraged to document: o Time spent in NICU o Time spent in skin-to-skin care o Time spent breast-feeding/pumping breastmilk o Weekly knowledge needs and skills learned o Experiences and feelings in online text/photo journal • Additional mFICare-specific We3health^™^ modules: o Rounds tab to record/retrieve notes and plan details o Recorded class content o Parent mentor advice and support messages o Textbot automated/customized knowledge, tips and encouragement

After completion of enrollment for the FCC group, the study was paused for two to three months at each site so the study team could provide in-person and online training to the volunteer “mFICare nurse champions” and alumni parent peer mentors. Additionally, approximately 80% of all other NICU nurses, nurse practitioners, physicians, therapists, and social workers received in-person and online in-service education specific to their roles. The training followed the Canadian FICare staff curriculum [20, 21].

After completion of training, parents and infants were enrolled in the mFICare group. In addition to all the FCC supports and services described above, the mFICare group received: parent group educational classes 2–5 times per week, following the Canadian FICare parent curriculum, with additional classes added based on local site interests [20, 21]; additional encouragement and expanded role for parents in direct infant caregiving (excluding ventilation management, intravenous fluid or intravenous medication administration); parent participation in weekday rounds; parent peer mentorship and an expanded version of the We3health™ app designed for the mFICare group (Table 1).

### Measures

Our primary infant outcome was the change in weight z-scores over the 21 days after study enrollment (study days 1 to 22). Weights on study day 29 and at discharge were also collected for sensitivity analyses. Weights were obtained from the infant medical record, and z-scores were calculated to standardize weights against gestational age-based norms [22]. A z-score change of zero indicates that an infant’s weight has a consistent percentile rank over time relative to preterm infant growth norms, change values greater than zero indicate an increasing percentile rank (i.e., weight gain that is faster than age-based norms), and change values less than zero indicate a decreasing percentile rank (i.e., weight gain that is slower than age-based norms).

Secondary infant outcomes included in this analysis were three common preterm morbidities recorded in the discharge summary (nosocomial infection, bronchopulmonary dysplasia [BPD], or retinopathy of prematurity [ROP]) and not receiving human milk feeds (HMF) at discharge. Information on the infant’s clinical course was collected from their medical records. We also recorded any adverse events involving parents in both cohorts.

mFICare intervention fidelity was monitored monthly to ensure that all families assigned to the mFICare group were being offered the opportunity to participate in each of the mFICare components. Monthly fidelity monitoring involved the research team documenting parent being offered and participating in different components of the mFICare intervention: acceptance of a peer mentor, degree of participation in weekday rounds (based on direct observation or information collected from the infant’s nurse), attendance at group classes (recorded by class facilitator), and use of the mFICare app. Results were regularly discussed with all NICU stakeholder groups (nursing, medicine, parent advisors, NICU leadership) within and across participating NICUs, and additional education, coaching, improvements to procedures and resource materials were provided at all sites throughout the project.

### Statistical analysis

Analyses were performed using R v4.1 (R Foundation, Vienna, Austria) and Stata v14.2 (StataCorp, College Station, TX). Descriptive statistics were calculated and comparisons by intervention group were performed using chi-square tests for categorical variables, analysis of variance for normally distributed continuous variables, and Mann-Whitney or Kruskal-Wallis tests for non-normally distributed continuous variables. For these group comparisons, *p* < 0.05 was considered statistically significant.

We compared 21-day change in weight z-scores between the 2 groups over the 3-week study period (change in z-scores measured on study days 1, 8, 15, and 22, using a linear mixed model with LME4 v1.1.27.1), testing an interaction term between group and post-baseline weight measurements [23]. We adjusted for additional covariates using a hybrid approach, forcing in known confounders of gestational age and study site and using backwards stepwise selection to retain covariates that contributed *p* < 0.1 to the final model from potential confounders. We additionally conducted sensitivity analyses adding in weights measured at study day 29, and at discharge.

Second, we conducted a per protocol analysis of 21-day change in weight z-scores, evaluating the intervention dose effects of 4 mFICare components during the same 21-day study period: whether the enrolled parent had a mentor; participated in weekday rounds (trichotomized: no dose (< 10%), low dose [10–79%], or high dose [≥ 80%]); attended group classes (trichotomized: no dose [0 classes], low dose [< 1/week], or high dose [≥ 1/week]; or regularly used the We3health™ app (trichotomized: no dose [< 1/week], low dose [1–3/week], or high dose [≥ 4/week]).

Third, we conducted intent-to-treat and per protocol analyses of the mFICare intervention effects on the secondary outcomes: nosocomial infection, BPD, ROP, and no HMF at discharge. We used logistic regression for these analyses because the secondary outcomes were dichotomous. We used the same procedure for inclusion of covariates as before, except that we did not include nosocomial infection, BPD or ROP as potential confounders (as they were now outcomes). For these secondary outcomes, which spanned the entire hospitalization, we used the mFICare intervention dose from enrollment to discharge.

mFICare group assignment and participation in the individual intervention components served as the reference groups for all regression analyses so that the statistical results highlight the increased risk of adverse outcomes with usual FCC compared with mFICare (rather than the decreased risk with mFICare compared with usual FCC). For the intention-to-treat analysis of our primary outcome, we used *p* < 0.05 for statistical significance, and for the secondary outcomes, we used a Bonferroni-corrected *p* < 0.0125. For the per protocol analysis, we noted results with a nominal *p* < 0.05, as none reached Bonferroni-corrected significance. Additional sensitivity analyses were conducted to evaluate the impact of including infants who were transferred to another hospital before being discharged home (*n* = 27); or infants who were hospitalized during the initial months of the COVID-19 pandemic (*n* = 26).

## Results

### Sample characteristics

**Fig. 1:**
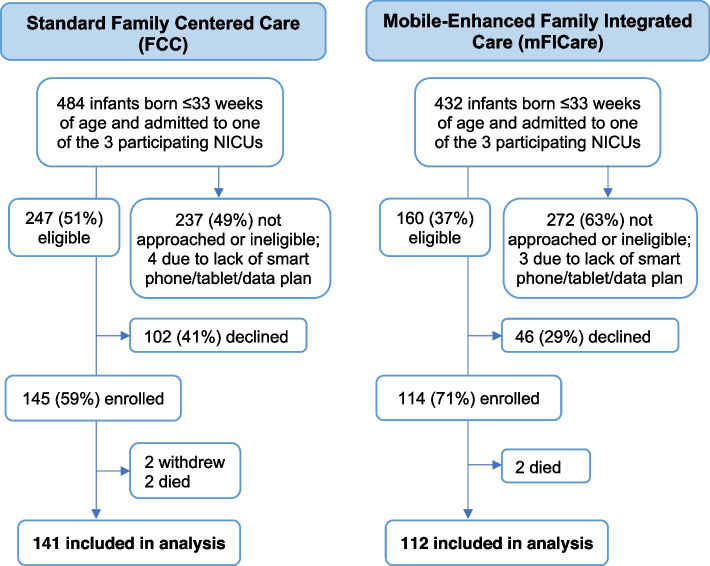
Participant enrollment by group

Participants were enrolled between April, 2017 and June, 2020. The final sample included 253 infants (Fig. 1).

Table 2 summarizes infant characteristics by intervention group. There were no significant differences in sample characteristics, length of stay or outcomes at discharge by intervention group. COVID-19 related hospital restrictions partially curtailed the intervention for 23% (n = 26) of the mFICare group. These infants did not differ from the rest of the sample on any characteristics in Table 2. In sensitivity analyses, only one finding was attenuated by their exclusion (described below). Additional sensitivity analyses indicated that including infants transferred to another hospital before being discharged home had minimal impact on study findings (data not shown). No parent-related adverse events related were reported in either group.

**Table 2:** Infant characteristics by intervention group (*N* = 253)

Characteristics	FCC (*n *= 141)	mFICare (*n* = 112)	*P*-value
**Demographic characteristics**			
NICU site, % (n)			0.52
Site A	37% (53)	44% (50)	
Site B	23% (32)	20% (22)	
Site C	40% (56)	36% (40)	
Female sex, % (n)	49% (69)	41% (46)	0.21
Race/ethnicity, % (n)			0.72
Asian	9% (13)	13% (15)	
Black	16% (22)	16% (18)	
Hispanic/Latino (any race)	38% (54)	37% (41)	
White	23% (32)	25% (28)	
Other or multiple race	10% (14)	5% (6)	
Unknown	4% (6)	4% (4)	
**Birth characteristics**			
Mean gestational age (GA), weeks	28.6 (2.8)	28.5 (2.5)	0.92
Gestational age group, % (n)			0.52
22–28 weeks	50% (71)	54% (61)	
29–33 weeks	50% (70)	46% (51)	
Mean birthweight, grams	1194 (470)	1182 (462)	0.85
Small for gestational age, % (n)	9% (13)	10% (11)	0.87
Outborn, % (n)	33% (46)	29% (32)	0.49
Multiple birth, % (n)	13% (18)	19% (21)	0.19
Mean Apgar score at 5 min	7.0 (1.8)	6.9 (2.1)	0.84
Apgar score ≥ 7, % (n)	72% (101)	64% (70)	0.18
**Clinical characteristics**			
Ventilation in NICU, % (n)	55% (77)	59% (66)	0.49
Any surgeries, % (n)	26% (36)	25% (28)	0.87
Median number of surgeries	0 (0–5)	0 (0–6)	0.92
Mean days on total parenteral nutrition	21.5 (2.1)	25.3 (2.8)	0.28
Intraventricular hemorrhage, % (n)	21.3% (30)	23.2% (26)	0.71
Necrotizing enterocolitis, % (n)	9.2% (13)	8.9% (10)	0.94
**Discharge characteristics**			
Mean length of hospital stay, days	74 (52)	84 (55)	0.14
Disposition, % (n)			0.39
Home	90% (127)	88% (99)	
Transferred to another hospital (lower intensity care)	10% (14)	13% (13)	
Discharged home or transferred with a respiratory device, % (n)	20% (28)	23% (26)	0.52
Discharged home or transferred with a feeding device, % (n)	19% (26)	24% (27)	0.28
**Study characteristics**			
Median postnatal age at study enrollment, days	15 (4–97)	19 (4-117)	0.09
Mean gestational age at enrollment, wks	31.8 (2.4)	32.2 (2.9)	0.25

Data are presented as means (SD), medians (IQR), or % (n). *P*-values are for independent t-tests, Mann-Whitney U tests, and chi-square tests, as appropriate

#### Intervention doses

All mFICare group participants were offered a parent mentor, participation in weekday clinical team rounds, weekly group classes, and unlimited use of the We3health™ mFICare version app. However, parents varied in their use of each intervention component. The frequencies of dose levels of each component are summarized in Table 3. Data collected through the app on parental presence and skin-to-skin care were found to be inconsistent among parents and over time. Some parents used the app daily; some used it initially, then decreased use over time; and some used it once or twice and then didn’t engage any further, despite encouragement. Given the inconsistent documentation of the parental presence and skin-to-skin data by parents or by staff in the electronic medical records, these data were not included in the analysis. In addition, based on input from parent advisors during the study planning and considering the often informal nature of parent mentoring (e.g., phone, text, email, social media chat), the amount of contact between participating parents and their mentors was not recorded.

**Table 3:** Doses of four intervention components for the 112 participants in the mFICare group

Intervention component	3-week study period % (n)	Entire hospitalization % (n)
**Paired with a parent mentor**	21% (24)	29% (32)
**Participation in clinical team rounds**		
Mean number participated per week (SD)	4.1 (2.1)	3.7 (2.0)
Median number participated per week (IQR)	4.3 (0–7)	4.0 (0–7)
Category		
No dose: 0–1 rounds	6% (7)	5% (6)
Low dose: 2–11 rounds	38% (43)	22% (25)
High dose: 12 or more rounds^a^	55% (62)	72% (81)
**Parent group educational classes**		
Mean number attended (SD)	3.5 (4.2)	6.5 (8.7)
Median number attended (IQR)	2 (0–11)	3 (0–30)
Category		
No dose: 0 classes	30% (34)	23% (26)
Low dose: 1–2 classes	28% (31)	21% (24)
High dose: 3 or more classes	42% (47)	55% (62)
**mFICare app (We3health™)**		
Mean number of app logins (SD)	10.4 (8.1)	16.3 (15.0)
Median number of app logins (IQR)	8 (0–27)	10 (0–54)
Category		
No dose: 0–3 app logins	19% (21)	16% (18)
Low dose: 4–11 app logins	44% (49)	38% (42)
High dose: 12 or more app logins	37% (42)	46% (52)

^a^At least 80% of all weekday clinical team rounds occurring in the 3-week initial intervention period

### Primary outcome: weight gain

**Fig. 2:**
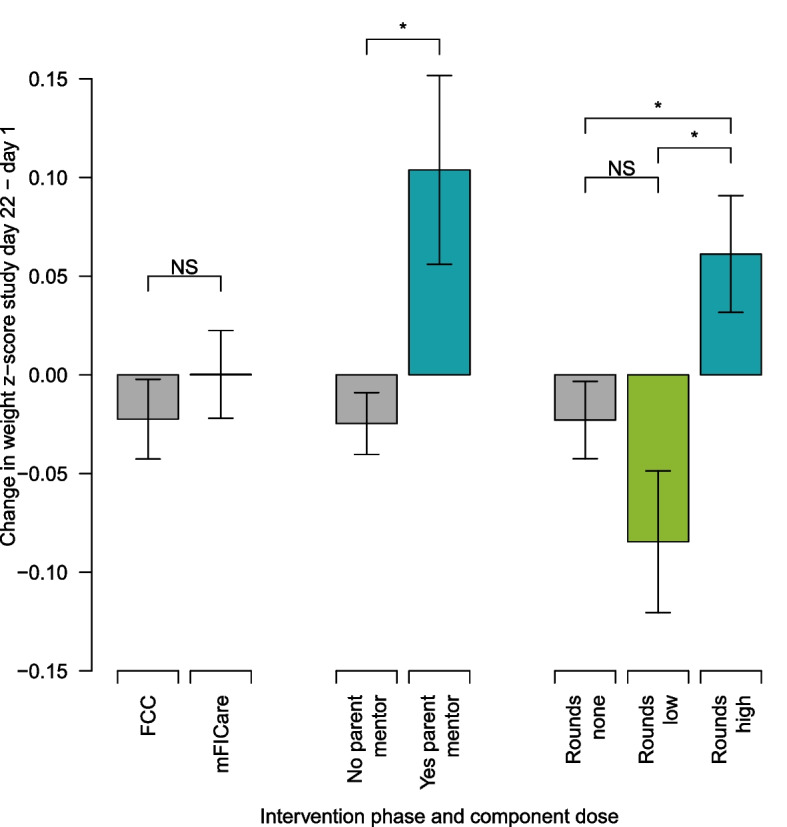
Weight gain by intervention group and mFICare component (paired with a parent mentor, participation in weekday rounds; adjusted for covariates). Bars are based on the linear mixed model, and error bars represent standard error; *indicates significantly more weight gain (P < 0.05); NS, not significant (*P* > 0.05)

Intention-to-treat: The final model indicated that 21-day change in weight z-scores did not differ by intervention group (Fig. 2; Table 4). To determine whether intervention effects required more than 3 weeks to emerge, we performed sensitivity analyses to model infant weight change over a 4-week study period (study day 1 to 29) and from enrollment to discharge, but no differences by intervention group were found (data not shown).

**Table 4:** Intention-to-treat evaluation of infant outcomes by intervention group

Outcome	FCC (*n* = 141)	mFICare (*n* = 112)			
**Primary: Weight gain**	**Mean (SD) {n}**	**Mean (SD) {n}**	***p*-value** ^**a**^	**Adjusted** **difference**^**c**^ **(95% CI) {n}**	***p*-value**
Change in weight z-score from study day 1 to 22	-0.028 (0.341) {124}^b^	-0.002 (0.449) {100}^b^	0.40	-0.023 (-0.082, 0.036) {250}	0.45
**Secondary: Morbidities and feeding**	**% (n)**	**% (n)**		**Adjusted** **OR**^**d−g**^ **(95% CI) {n}**	
Nosocomial infection	14.9% (21)	7.1% (8)	0.055	2.6 (1.0, 6.7) {253}	**0.049**
Bronchopulmonary dysplasia (BPD)	21.6% (30)	24.1% (27)	0.64	0.5 (0.2, 1.2) {249}	0.15
Retinopathy of prematurity (ROP)	34.8% (49)	33.0% (37)	0.78	1.5 (0.7, 3.1) {253}	0.42
No human milk feeding at discharge	64.3% (90)	56.3% (63)	0.19	1.5 (0.9, 2.6) {243}	0.28

*P*-values < 0.05 are in bold. mFICare is the reference group for regression analyses. Odds ratios (OR) < 1 indicate better outcomes in the FCC group than in the mFICare group; ORs > 1 indicate worse outcomes in the FCC group

^a^*P*-values for unadjusted comparisons of change in weight z-scores (Mann-Whitney U tests) and morbidity and feeding outcomes (chi-square tests) by intervention group

^b^Sample size is limited to the 224 infants with weight z-scores s at both study day 1 and 22. Negative z-score changes indicate that infant weight had a decreasing percentile rank over time (i.e., weight gain was slower than preterm infant growth norms)

^c^Weight gain model (mixed linear) adjusts for site, gestational age at birth, small for gestational age, necrotizing enterocolitis, and morbidity count (a proxy of clinical course complexity, defined as the total of five common preterm morbidities during the NICU stay [nosocomial infection, BPD, ROP, necrotizing enterocolitis, and intraventricular hemorrhage], trichotomized as 0, 1 or 2–5). Negative difference indicates that FCC group gained less weight than the mFICare group

^d^Infection model (logistic) adjusts for site, gestational age at birth, and any ventilation

^e^BPD model (logistic) adjusts for site, gestational age at birth, and any surgery

^f^ROP model (logistic) adjusts for site, gestational age at birth, and small for gestational age

^g^Human milk feeding model (logistic) adjusts for site, gestational age at birth, Hispanic ethnicity, and BPD

Per protocol analysis of intervention components: Infants whose parents had not been paired with a parent mentor or attended 11 or fewer clinical team rounds during the 3-week study period lost nominally more weight relative to age-based norms than infants whose parents had a mentor or attended ≥ 12 rounds (mentor group difference = -0.128, 95% CI: -0.227, -0.030; 0–1 vs. ≥12 rounds group difference = -0.084, 95% CI: -0.154, -0.015; 0–1 rounds group difference = 0.061, 95% CI: -0.018, 0.141; 2–11 vs. ≥12 rounds group difference = -0.146, 95% CI: -0.237, -0.055; Fig. 2; Table 5). The effect of rounds was attenuated if parents affected by COVID-19 related hospital restrictions (including discontinuation of in-person and remote parent participation on rounds) were excluded from the analysis (data not shown).

**Table 5:** Per protocol analysis of intervention components

Infant outcome Intervention component	Adjusted group difference^b^ or OR^c^ (95% CI)	*p*-value
**Weight gain during 3-week study period** (*n* = 250)		
** Paired with a parent mentor**		
Yes	reference	
No	-0.128 (-0.227, -0.030)	**0.011**
** Participating in clinical team rounds**		
12 or more rounds	reference	
2–11 rounds	0.062 (-0.018, 0.142)	0.13
0–1 rounds	-0.084 (-0.154, -0.015)	**0.018**
** Attending parent group educational classes**		
3 or more classes	reference	
1–2 classes	-0.000 (-0.090, 0.090)	> 0.99
0 classes	0.009 (-0.067, 0.085)	0.82
** Using the mFICare app**		
12 or more app logins	reference	
4–11 app logins	0.031 (-0.045, 0.106)	0.42
0–2 app logins	0.050 (-0.031, 0.130)	0.23
**Infection during NICU hospitalization** (*n* = 253)		
** Having a parent mentor**		
Yes	reference	
No	7.9 (0.7, 66.7)	0.059
** Participating in clinical team rounds**		
2 or more rounds^**a**^	reference	
0–1 rounds	2.9 (1.0, 9.1)	**0.043**
** Attending parent group educational classes**		
1 or more classes^**a**^	reference	
0 classes	3.8 (1.2, 14.3)	**0.016**
** Using the mFICare app**		
12 or more app logins	reference	
4–11 app logins	2.1 (0.5, 8.1)	0.30
0–3 app logins	1.8 (0.6, 5.6)	0.30

*P*-values < 0.05 are in bold. Other secondary outcomes were not associated with use of the intervention components

^a^Due to small cell counts in the infection models, low and high doses were combined both for rounds and for parent group classes, and exact logistic regression was used

^b^Weight gain models use intervention doses during the 3-week study period and are reported as the group difference in weight gain (change in weight z-scores) adjusted for site, gestational age at birth, small for gestational age, necrotizing enterocolitis, and morbidity count (a proxy of clinical course complexity, defined as the total of five common preterm morbidities during the NICU stay [nosocomial infection, BPD, ROP, necrotizing enterocolitis, and intraventricular hemorrhage], trichotomized as 0, 1 or 2–5)

^c^Infection models (logistic) use intervention dose from study enrollment to hospital discharge and are reported as odds ratios adjusted for site, gestational age at birth, and any ventilation

#### Secondary outcomes: morbidities and human milk feeding

**Fig. 3:**
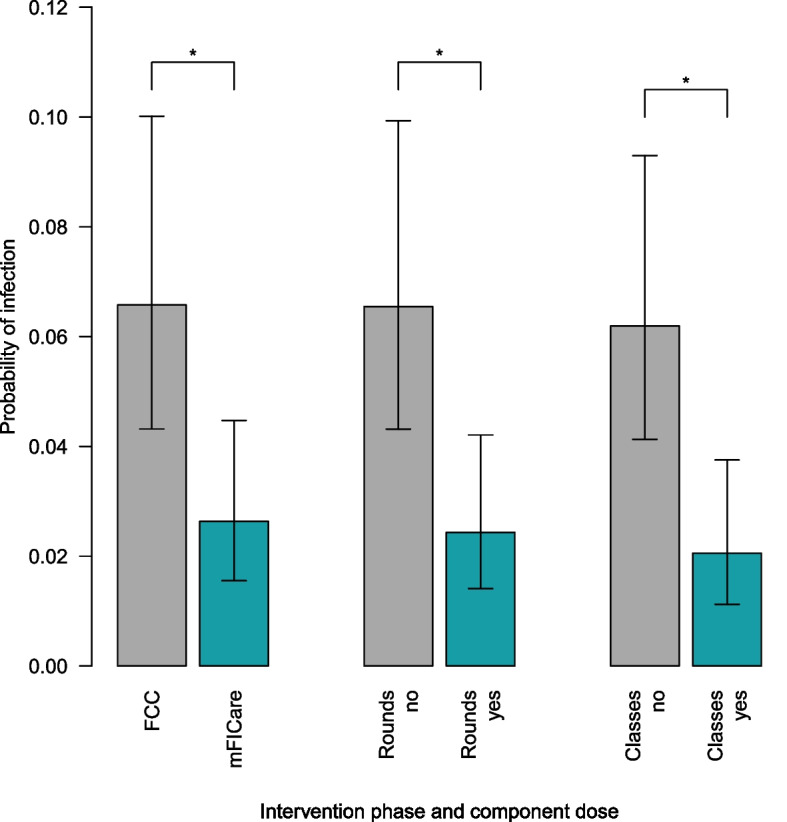
Infection rates by intervention group and mFICare component (participation in weekday rounds and group educational classes; marginal mean effects adjusted for covariates), and error bars represent standard error. *Significantly lower risk than standard care (FCC) or not receiving intervention component

Morbidities: The odds of acquiring a nosocomial infection were 2.6 times higher in the usual FCC group compared with the mFICare group, adjusting for relevant covariates (OR = 2.6, 95% CI: 1.0, 6.7; *p* < 0.05; Fig. 3), but none of the other morbidities differed by intervention group (Table 4). Infants of parents who participated in 0–1 clinical team rounds during their hospitalization had 2.9 times higher odds of acquiring a nosocomial infection (OR = 2.9, 95% CI: 1.0, 9.1; *p* < 0.05; Table 5; Fig. 3) than infants of parents who participated in two or more clinical team rounds. Infants whose parents did not participate in any group classes had 3.8 times higher odds of acquiring a nosocomial infection (OR = 3.8, 95% CI: 1.2, 14.3, *p* < 0.05; Table 5; Fig. 3) than infants of parents who participated in any of the group classes.

Feeding: To determine whether the mFICare intervention had any impact on HMF at discharge, regression models were fit to assess the associations between intervention variables (study group and dose of each mFICare component) and the infant not being discharged on HMF. The proportion of infants not receiving HMF at discharge did not differ by intervention group (Table 4) or by use of any mFICare intervention component (data not shown).

## Discussion

In this quasi-experimental study to investigate the effects of the mFICare program compared to usual FCC in US NICUs on infant clinical outcomes, our intention-to-treat analyses showed that infant weight gain did not differ between groups. However, we found that infants in the usual FCC group had 2.6 times higher odds of acquiring nosocomial infection during their NICU stay than infants in the mFICare group. In our per protocol analyses, we found that if parents engaged in the mFIcare program as indicated by being paired with a parent mentor and/or regularly participating in rounds, infants had improved weight gain by 21 days after enrollment compared to if their parents had not been paired with a parent mentor or had not participated in weekday rounds. Similarly, we also found that when parents engaged in the mFICare program by participating in rounds or classes, infants had between approximately 2.9 or 3.8 times lower odds of acquiring a nosocomial infection compared to if their parent had not participated in at least 2 weekday rounds or at least one group class during the 3-week study period, respectively. These results suggest that the mFICare program may have beneficial effects on infant clinical outcomes. While acknowledging the importance of the interaction and mutual reinforcement of the program components, our results provide a preliminary indication that parental engagement in mentorship, parent participation in clinical team rounds, and parent group classes, showed promise for improving infant outcomes in this study. Further research will be needed.

Previous studies have found decreased infection rates in infants receiving FICare compared with FCC [10, 13]. Studies have demonstrated the benefits to patient care quality and safety of including parents as active members in clinical rounds [24] and providing parents with knowledge and emotional/practical support through individual peer parent mentorship and group support/education [25, 26]. FICare is a model of NICU care delivery designed to support greater parent engagement in the care of their infant in the NICU. The improved infant outcomes found in previous studies are attributed to the increased parent engagement across the continuum of caregiving and shared decision-making, not to any specific component of the program itself. It is challenging for bedside care providers to record parent participation in care, and even the use of a mobile app to support parents recording their own participation was incomplete. However, our research team collected detailed information from nurses on how often parents were present in rounds, how often they attended education sessions and how many were connected with parent mentors as measures of model fidelity. Although participation in these activities as part of mFICare is likely mutually reinforcing, the differential effects in our study suggest that parents may have distinct needs or preferences for different types of involvement or support. Engagement of parents as full partners in their infant’s healthcare is visibly demonstrated through their active inclusion in rounds where their infant’s clinical progress is reviewed, and care plan decisions are made. We speculate that these FICare components may impact infant outcomes through activation of parents in infant caregiving, particularly with respect to developmental care for infants [27, 28], and greater knowledge and assertiveness, specifically regarding hygiene and feeding [29]. Of note, we did not find a specific effect of the mobile app on infant outcomes, as was previously reported [12]. However, the We3health™ app may have contributed indirectly by increasing parent participation in other FICare activities (e.g., rounds, classes, mentor contact). Finally, no adverse events related to parental participation in mFICare were reported. Future studies are needed to confirm these findings in the US context and further explore mechanisms of action.

In contrast to the previous multi-site cluster randomized trial of the FICare program [11], we found no intervention group effect on either weight gain or HMF compared with usual FCC care. This may be due to differing inclusion criteria, with the infants in our study being overall smaller, sicker, more racially/ethnically diverse, and more likely to be outborn, and their parents becoming involved in the mFICare program later in the NICU stay. Infants in our study also required more surgery, ventilatory support, and feeding support at discharge than the prior international trial. It is also possible that we did not achieve a sufficient dose of the mFICare intervention, or succeed in increasing parents’ active participation in caregiving, because we did not require parents to be present in the NICU for a minimum number of hours per weekday as in the previous trial. We also were unable to provide parents at all sites with free parking, transportation, or childcare for siblings during parents’ time in hospital, which may have curtailed their mFICare participation. Moreover, FCC practices at all three of the study sites were well developed, with many of the environmental and staff knowledge and attitude practices in place [28]. Thus, the main differences between the intervention and usual care groups were the components of parent mentorship, participation in rounds and parent group education as well as in the consistency of tracking and monitoring parental engagement in their infant’s care.

Our study contributes to the growing research on the benefits of supporting and partnering with parents of preterm and ill infants. Our study is unique in the level of detail we captured on parental participation in the main mFICare components, allowing us to identify the components that most likely influenced the selected outcomes. Although our approach of analyzing the individual components of the mFICare bundle may overestimate their impact if they were to be implemented separately, it does provide a preliminary indication of which components are most strongly associated with which outcomes and what might be a minimal effective level of engagement for each. These findings provide crucial implementation guidance to clinical teams, such as which elements of the intervention bundle they may want to emphasize first or where to focus quality improvement efforts to promote parental engagement. Nonetheless, future research is needed to determine if there is a dose effect of the number of rounds a parent participates in or the number of classes they attend, as well as the relative importance of specific class content (e.g., developmental care) [27, 28]. These findings can inform implementation strategies to enhance fidelity, quality, and dose of the mFICare components in future research and quality improvement initiatives.

Our study had several limitations in design and implementation. First, there may be selection bias because of the non-random design. Although we observed no demographic or clinical differences between the groups, unmeasured changes in parental presence, parent support, or clinical practices during the two enrollment periods could have influenced the outcomes. Second, not all families in the mFICare group received the full intervention dose. This may be due to inconsistencies in mFICare practices across large staff groups at the three sites, despite offering extensive training and support, as well as enrollment later in the hospital admission (or after transfer from another NICU). Individual parent circumstances and preferences, particularly with respect to financial or social barriers, likely limited some parents’ participation. Thus, the negative findings of the intention-to-treat analyses may be due to insufficient intervention delivery rather than the intervention’s lack of impact. Moreover, family presence, participation in infant caregiving and decision-making and family support services were all curtailed by the hospitals’ responses to the COVID-19 pandemic, reducing intervention dose for some participants. Third, the study excluded non-English-speaking families, limiting generalizability. Finally, we examined only a few possible infant outcomes and future longitudinal follow-up studies are needed as FICare’s greatest impact may occur after discharge in improved infant neurodevelopment and parent mental health [8, 10].

Despite the limitations, we successfully delivered the mFICare program to a racially/ethnically diverse cohort of families in three types of NICUs serving different populations. Our research highlights the need for improvement in hospital data systems to do a better job in aggregating information on parental presence and involvement in caregiving and decision-making for research and quality improvement purposes. Unfortunately, most hospital clinical data systems are poorly designed to accurately collect and aggregate data on parental presence and involvement. Despite substantial nursing time spent documenting such activities, the inconsistency in documentation locations, terminology and missingness mean that the data are of poor quality. Redesign of hospital information systems and integration of available technology are urgently needed to address the data gaps, reduce nursing data entry burden and improve usability of data on parent presence and participation in caregiving and decision-making. Design of apps for parents must also be improved to reduce parent data entry burden and improve positive feedback to encourage ongoing involvement and parenting skill acquisition and mastery.

Further research of the FICare model is needed, using implementation science methods [30] to address the barriers and bottlenecks to FICare implementation in the US context. This includes greater focus on structural barriers to parental presence and active involvement in their infant’s care, addressing paid family leave, childcare for siblings, costs of transportation, parking, food, overnight accommodation, and other out-of-pocket expenses. Institutional barriers to transforming the NICU culture to one that fully supports and promotes full parental partnership in care include staff education, attitudes and beliefs, power hierarchy, and workload [5, 31]. Until these structural issues are addressed, neither FCC nor mFICare will achieve their full potential to improve infant and family outcomes.

In summary, we found promising indications that mFICare may have direct benefits on infant outcomes such as weight gain and nosocomial infection. Future research using implementation science designs are needed to optimize intervention delivery and determine acute and long-term infant and family outcomes.

## Data Availability

Deidentified data will be shared upon reasonable request directed to Linda S. Franck (linda.franck@ucsf.edu) from qualified investigators beginning 6 months and ending 5 years after publication.

## Abbreviations

BPD: Bronchopulmonary Dysplasia
FCC: Family-Centered Care
FICare: Family Integrated Care
mFICare: mobile-enhanced Family Integrated Care
NICU: Neonatal Intensive Care Unit
ROP: Retinopathy of Prematurity
US: United States

## References

[1] Franck LS, O’Brien K (2019). The evolution of family-centered care: From supporting parent‐delivered interventions to a model of family integrated care. Birth Defects Res.

[2] World Health Organization. Survive and thrive: transforming care for every small and sick newborn. [Internet]. World Health Organization; Geneva; 2019 [Cited 2022 Apr 11]. Available from: https://www.who.int/publications/i/item/9789241515887.

[3] Davidson JE, Aslakson RA, Long AC, Puntillo KA, Kross EK, Hart J (2017). Guidelines for family-centered care in the neonatal, pediatric, and adult ICU. Crit Care Med.

[4] Dhurjati R, Sigurdson K, Profit J (2019). Patient- and family-centered care as a dimension of quality. Am J Med Qual.

[5] Franck LS, Bisgaard R, Cormier DM, Hutchison J, Moore D, Gay C (2022). Improving family-centered care for infants in neonatal intensive care units: Recommendations from frontline healthcare professionals. Adv Neonatal Care..

[6] Sigurdson K, Profit J, Dhurjati R, Morton C, Scala M, Vernon L (2020). Former NICU families describe gaps in family-centered care. Qual Health Res.

[7] Moreno-Sanz B, Montes MT, Antón M, Serrada MT, Cabrera M, Pellicer A (2021). Scaling up the family integrated care model in a level IIIC neonatal intensive care unit: A systematic approach to the methods and effort taken for implementation. Front Pediatr.

[8] Synnes AR, Petrie J, Grunau RE, Church P, Kelly E, Moddemann D (2022). Family integrated care: very preterm neurodevelopmental outcomes at 18 months. Arch Dis Child Fetal Neonatal Ed.

[9] Benzies KM, Aziz K, Shah V, Faris P, Isaranuwatchai W, Scotland J (2020). Effectiveness of Alberta Family Integrated Care on infant length of stay in level II neonatal intensive care units: a cluster randomized controlled trial. BMC Pediatr.

[10] Hei M, Gao X, Li Y, Gao X, Li Z, Xia S (2021). Family integrated care for preterm infants in China: A cluster randomized controlled trial. J Pediatr.

[11] O’Brien K, Robson K, Bracht M, Cruz M, Lui K, Alvaro R (2018). Effectiveness of Family Integrated Care in neonatal intensive care units on infant and parent outcomes: a multicentre, multinational, cluster-randomised controlled trial. Lancet Child Adolesc Health.

[12] Banerjee J, Aloysius A, Mitchell K, Silva I, Rallis D, Godambe SV (2020). Improving infant outcomes through implementation of a family integrated care bundle including a parent supporting mobile application. Arch Dis Child Fetal Neonatal Ed.

[13] van Veenendaal NR, van der Schoor SRD, Heideman WH, Rijnhart JJM, Heymans MW, Twisk JWR (2020). Family integrated care in single family rooms for preterm infants and late-onset sepsis: a retrospective study and mediation analysis. Pediatr Res.

[14] Moe AM, Kurilova J, Afzal AR, Benzies KM (2022). Effects of Alberta family integrated care (FICare) on preterm infant development: Two studies at 2 months and between 6 and 24 months corrected age. J Clin Med.

[15] Patel N, Ballantyne A, Bowker G, Weightman J, Weightman S (2018). Family integrated care: Changing the culture in the neonatal unit. Arch Dis Child..

[16] Franck LS, Waddington C, O’Brien K (2020). Family integrated care for preterm infants. Crit Care Nurs Clin North Am.

[17] Montez K, Thomson S, Shabo V (2020). An opportunity to promote health equity: National paid family and medical leave. Pediatrics.

[18] Kyle MA, Frakt AB (2021). Patient administrative burden in the US health care system. Health Serv Res.

[19] Franck LS, Kriz RM, Bisgaard R, Cormier DM, Joe P, Miller PS (2019). Comparison of family centered care with family integrated care and mobile technology (mFICare) on preterm infant and family outcomes: a multi-site quasi-experimental clinical trial protocol. BMC Pediatr.

[20] O’Brien K, Bracht M, Robson K, Ye XY, Mirea L, Cruz M (2015). Evaluation of the Family Integrated Care model of neonatal intensive care: a cluster randomized controlled trial in Canada and Australia. BMC Pediatr.

[21] Implementing Family Integrated Care [Internet]. 2021 [cited 2021 Sep 21]. Available from: http://familyintegratedcare.com/implementing-ficare/.

[22] Fenton TR, Sauve RS (2007). Using the LMS method to calculate z-scores for the Fenton preterm infant growth chart. Eur J Clin Nutr.

[23] Bates D, Mächler M, Bolker B, Walker S (2015). Fitting linear mixed-effects models using lme4. J Stat Soft.

[24] Davidson JE (2013). Family presence on rounds in neonatal, pediatric, and adult intensive care units. Ann Am Thorac Soc.

[25] Hunt H, Whear R, Boddy K, Wakely L, Bethel A, Morris C (2018). Parent-to-parent support interventions for parents of babies cared for in a neonatal unit-protocol of a systematic review of qualitative and quantitative evidence. Syst Rev..

[26] Steinhardt A, Hinner P, Kühn T, Roehr CC, Rüdiger M, Reichert J (2015). Influences of a dedicated parental training program on parent-child interaction in preterm infants. Early Hum Dev.

[27] European Foundation for the Care of Newborn Infants. Infant- & family-centred developmental care [Internet]. European Standards of Care for Newborn Health. [cited 2022 Aug 11]. Available from: https://newborn-health-standards.org/standards/standards-english/infant-family-centred-developmental-care/.

[28] Consensus Committee of the Standards, Competencies, and Best Practices for Infant and Family-Centered Developmental Care in the Intensive Care Unit. Developmental Care Standards for Infants in Intensive Care [Internet]. NICU Recommended Standards. [cited 2022 Aug 11]. Available from: https://nicudesign.nd.edu/nicu-care-standards/.

[29] Lyndon A, Wisner K, Holschuh C, Fagan KM, Franck LS (2017). Parents’ perspectives on navigating the work of speaking up in the NICU. J Obstet Gynecol Neonatal Nurs.

[30] Handley MA, Lyles CR, McCulloch C, Cattamanchi A (2018). Selecting and improving quasi-experimental designs in effectiveness and implementation research. Annu Rev Public Health.

[31] Zanoni P, Scime NV, Benzies K, McNeil DA, Mrklas K, Alberta FICare in Level II NICU Study Team, et al. Facilitators and barriers to implementation of Alberta family integrated care (FICare) in level II neonatal intensive care units: a qualitative process evaluation substudy of a multicentre cluster-randomised controlled trial using the consolidated framework for implementation research. BMJ Open. 2021;11(10)e054938. 10.1136/bmjopen-2021-054938 PMC8524282 34663673

